# Melon Seed Oils as Rich Sources of Linoleic and Oleic Acids: An Upcycling Approach for Food Applications

**DOI:** 10.1021/acsomega.6c05978

**Published:** 2026-09-07

**Authors:** Liliane Fernandes do Nascimento, Joselito Brilhante Silva, Laércio Galvão Maciel, Diogenes Henrique Abrantes Sarmento, Maria Aparecida Liberato Milhome

**Affiliations:** † 537144 Instituto Federal de Educação, Ciência e Tecnologia do Ceará - Campus Limoeiro do Norte (IFCE), Pós-Graduação em Tecnologia de Alimentos (PGTA), Limoeiro do Norte, Ceará 62930-000, Brasil; ‡ 74384 Universidade Federal Rural do Semi-árido (UFERSA), Mossoró, Rio Grande do Norte 59625-900, Brasil

## Abstract

Melon (*Cucumis melo* *L*.) is a fruit from the Cucurbitaceae family that is very popular due to its pleasant taste. The fruit is used in industry to produce juices and pulps, and the seeds are generally discarded as waste. To apply the concepts of circular economy (upcycling) and enable the use of this agro-industrial waste, this work presents a study on the use of oil extracted from the seeds of three melon varieties supplied by a company operating in the Jaguaribe Valley, Ceará, Brazil. The main objective of this study was to comparatively evaluate the fatty acid composition of seed oils from three melon cultivars (yellow melon, Piel de Sapo melon, and cantaloupe melon) as a source of fatty acids for food purposes and with gastronomic potential. Gas chromatography (GC) analysis of the fatty acid profile indicated the presence of linolenic acid (ω-3), linoleic acid (ω-6), and oleic acid (ω-9) in the oils obtained. PCA and heat map analyses showed distinct characteristics in the fatty acid profile for each melon variety: the yellow and Piel de Sapo melons showed a higher content of saturated fatty acids (SFA) and less diversity of unsaturated fatty acids (UFA); the Piel de Sapo melon showed a predominance of oleic and linoleic acids; the cantaloupe melon showed the highest levels of linolenic acid among the three cultivars. This study is relevant to the food industry because it presents an unconventional and sustainable source of essential fatty acids.

## Graphical abstract

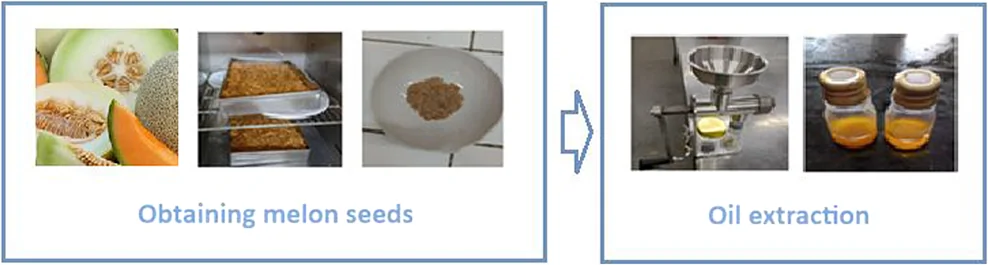

## Introduction

Melon (*Cucumis melo L*.) is a seasonal fruit belonging to the Cucurbitaceae family and is known and widely cultivated throughout the world. The fruit has great economic and social importance in Brazil, especially in the Northeast region. The states of Rio Grande do Norte and Ceará are the largest producers, accounting being responsible for over 99% of the national production destined for export, mainly to the European market.^1^

In 2023, the value of melon exports in Brazil was more than US$189 million and Ceará accounted for 34.60% of these exports with a value of more than US$65 million. The Northeast region stands out in melon production, with Rio Grande do Norte (US$ 107,172.55), Bahia (US$ 20,304.21), and Ceará (US$ 18,319.24) among the main melon-producing states^2^ (average 2023 exchange rate: BRL 4.99 = USD 1.00). The fruit is cultivated in many countries and has high financial value worldwide all due to its adaptation to climate and various soil types. Melon varieties (*Cucumis melo L*.) have high economic value because their production has been promoted in many regions. while a large portion of the fruit is discarded.^3^

The market for premium melons in the country is developing due to consumer preference and the growing importance of differentiated cultivars.^4^ In Brazil, the best-known and most appreciated melons belong to the inodorus group. yellow type. with a long post-harvest shelf life. The “Valenciano” cultivar and its selections Amarelo, Amarelo CAC, and Eldorado 300 are the most cultivated. Other varieties and hybrids of so-called premium melons, such as Cantaloupe, Galia and Orange Flesh, whose main destination is the export market, are also being introduced.^4^

In general, during the processing and consumption of *Cucumis melo L*. the inedible parts such as seeds and peels are commonly discarded. According to Jorge *et al.* ^5^ melon belongs to the Cucurbitaceae family and is consumed worldwide. Its seeds, which represent about 10% of the total fruit mass, and are generally discarded as agricultural waste. Melon seed oil has several beneficial properties such as antibacterial and antioxidant activities, and has been investigated for its potential to reduce blood cholesterol levels, effects that may be associated with the presence of biologically active compounds.^6^

Melon seed oil has great potential as a functional ingredient.^7–9^ Zhang *et al.* ^9^ propose the use of melon seeds as an alternative source for vegetable oil extraction, potentially generating employment and income while adding socioeconomic value to this underutilized material. Recent research has also highlighted the potential use of different vegetable oils in gastronomy, contributing to sensory quality and culinary versatility.^10,11^ The use of melon seeds as a promising and unconventional source of vegetable oil can be a good example of increasing the value of fruit by-products.^12,13^

In the food sector, the circular economy has gained prominence as a production and consumption model that values sustainability focusing on the reduction, reuse, recovery, and recycling of materials and energy. In practice, the circular economy implies reducing waste and promoting the efficient use of resources.^14^ Upcycling, also known as creative reuse, is the process of using seemingly useless products, waste and by-products to create new products.^15,16^ It is a strategy that reuses materials that would otherwise be discarded but still have potential value. The difference between recycling and upcycling is that while recycling transforms waste into raw materials, upcycling goes further, transforming it into a final product.

This study suggests utilizing non-conforming fruits for export by extracting oil from melon seeds and using this oil in human food, for example, in salad dressings. As a result, fulfilling the principles of circular economy, upcycling, and sustainable development contributes to increased revenue for fruit processing companies.

## Materials and Methods

### Sampling and Extraction of Oil from Melon Seeds

Melon seed samples of 3 different cultivars, Yellow melon, Piel de Sapo melon and Cantaloupe melon were provided by a company operating in the Jaguaribe Valley, Ceará, Brazil in 2023.

The sample preparation procedure was carried out at Federal Institute of Educaion, Science and Technology of Ceará – IFCE Campus Limoeiro do Norte, Ceará, Brazil, according to the steps described in the flowchart presented in Figure 1. After separation, cleaning, and drying of the seeds, oil extraction was performed using a clean, nontoxic, solvent-free method. The chosen method was mechanical pressing using a manual extractor to obtain oil from melon seeds. This equipment consists of a continuous screw that crushes the seeds under heating, as shown in Figure 1.

**Figure 1:**
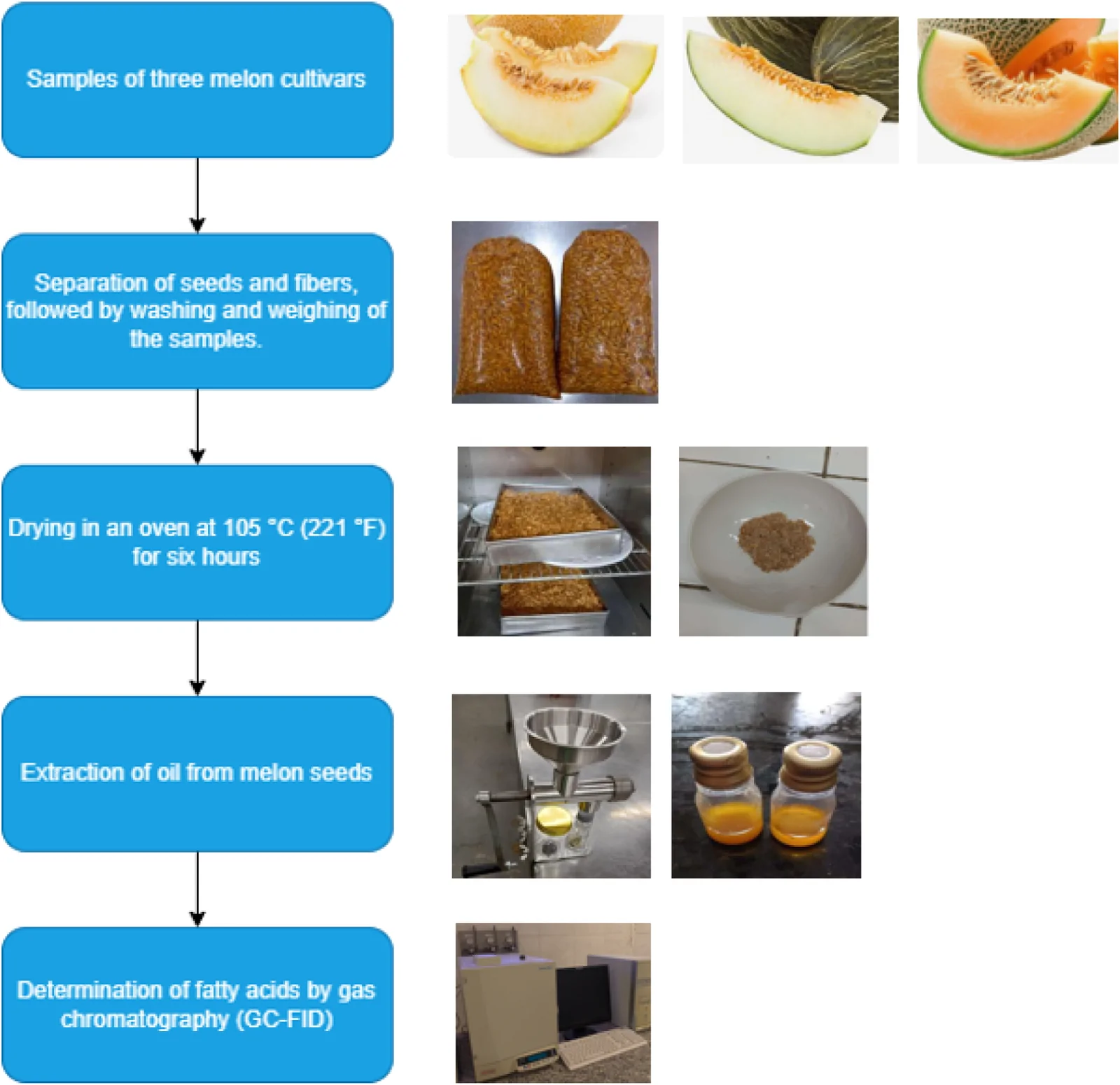
Flowchart of the experimental procedure.

To determine the oil yield for the three types of melon, the yield was calculated based on the amount of oil, in grams, divided by the mass (g) of sample used. This calculation was performed according to eq 1

1 $$\mathrm{Extracted oil content}\;(\%)=\left(m_{\mathrm{oil}}{/m}_{\mathrm{sample}}\right)\times 100$$

where m_oil_ is the oil mass (g) and m_sample_ is the total mass (g) of the sample.

### Fatty Acid Analysis by GC-FID

The determination of fatty acids from melon seed oil was carried out according to the Instituto Adolfo Lutz^17^ methodology using a gas chromatograph (model Focus, Thermo Electron Corporation) with a flame ionization detector (GC-FID). Fatty acid methyl esters were obtained from fatty acid and glycerol esters of oils and fats by transesterification reaction in a basic cold medium and subsequently analyzed by gas chromatography.

Initially, approximately 100 mg of the sample were weighed into a 20 mL centrifuge tube with a stopper. Then, 2 mL of *n*-hexane and 0.2 mL of methanolic KOH solution (2M) were added, followed by vortexing (30 seconds). After the reaction time, around 2 min, 3 mL of saturated sodium chloride solution were added to the tube for the partitioning step. After separation, the upper phase (organic phase) was transferred to a flask for subsequent analysis by gas chromatography.^17^

Determination of fatty acids was performed using GC-FID with a 100 m × 0.25 mm (i.d.) × 0.20 μm capillary column (SP-2560), using Nitrogen carrier gas (1 mL/min). The split injector (1:10) at a temperature of 250 °C was used with a split/splitless liner, split flow rate of 10 mL/min (3 mL/min purge flow) and an injection volume of 1 μL. The total run time was 49.50 min, with the column temperature starting at 100 °C, increasing to 220 °C at a rate of 4.0 °C/min and maintained for 5 min, then increasing to 250 °C at a rate of 4.0 °C/min and maintained for 7 min. The detector used was the FID, with a temperature of 260 °C and a solvent delay of 10 min. The flow ratio between the detector gases hydrogen, make-up (nitrogen), and synthetic air was 1:1:10, respectively.

Quantification was performed using the normalization method and component Fame Mix fatty acid standard (SUPELCO) for the identification of retention times

### Statistical Analysis

Data were analyzed using Microsoft Office Excel 2016 (Microsoft Inc., USA), R (v. 4.5.2), Jamovi 2.66 and OriginPro 2022. The results were subjected to analysis of variance (ANOVA), followed by Tukey’s test (p < 0.05) to detect differences among the results. Principal component analysis (PCA) was applied to reduce data dimensionality and identify clusters in the physicochemical composition among the different cocoa clones. The data were standardized (z-score), and varimax orthogonal rotation was applied to optimize the interpretability of the extracted factors, maximizing the variance of the factor loadings. Sample scores were projected onto the factorial planes defined by the first two principal components (PC1 and PC2), which accounted for the majority of the data variance. The selection of components was based on the eigenvalue greater than 1 criterion (Kaiser) and the analysis of cumulative explained variance, ensuring the retention of components that preserved most of the information from the original data.

## Results and Discussion

### Melon Seed Oil Yield Analysis

Table 1 presents the mean and standard deviation of the oil yields obtained from the seeds of the three melon cultivars using manual mechanical pressing. The oil yields from Yellow, Piel obtained from Yellow, Piel de Sapo, and Cantaloupe melon seeds were 4.90 ± 0.56, 9.98 ± 0.82, and 5.03 ± 0.80, respectively. Significant differences in oil yield were observed among the cultivars (p < 0.05). Tukey’s test showed that Yellow melon (4.90 ± 0.56%) and Cantaloupe melon (5.03 ± 0.80%) did not differ significantly from each other, whereas Piel de Sapo melon (9.98 ± 0.82%) showed a significantly higher yield than the other two cultivars. Thus, manual pressing of melon seed oil demonstrated yields ranging from 4.90% to 9.98%. The Piel de Sapo melon variety showed the best yield (9.98 ± 0.82a).

**Table 1:** Comparative Study of Oil Yield from the Seeds of Three Melon Cultivars Obtained by Manual Pressing (in This Study) with the Soxhlet Extraction Method^a^

	Extraction yield (%)	
Melon cultivars	Manual press (this study)	Soxhlet^18^
Yellow	4.90 ± 0.56b	28.47 ± 0.10
*Piel de Sapo*	9.98 ± 0.82a	26.41 ± 0.09
Cantaloupe	5.03 ± 0.80b	29.00 ± 0.00

a Values are expressed as mean ± standard deviation (n = 3). Different lowercase letters within the manual-pressing column indicate significant differences among cultivars according to Tukey’s test (p < 0.05). Identical letters indicate no significant difference.

One strategy to improve extraction yield would be to combine thermal pre-treatment (controlled roasting) with fine grinding and proper moisture adjustment prior to pressing. Pretreatment may be particularly suitable for manual pressing systems because it can reduce oil viscosity and promote disruption of cellular structures. Although enzymatic treatment is highly efficient in laboratory settings, it requires strict pH control and long incubation periods, making it unfeasible for purely manual processes.

Table 1 also compares the yields obtained by manual pressing in the present study with values reported for Soxhlet extraction. The method using hexane as a solvent yields significantly higher yields than mechanical pressing extraction, probably due to the nonpolar nature of the fatty acids present in the oil composition and, therefore, to intermolecular forces similar to those of the solvent. However, this method has the disadvantage of using a solvent, as there is currently a search for environmentally friendly and solvent-free methods, which has been encouraged in the scientific community

The results represent the mean ± standard deviation of the analysis performed in triplicate. A. B: Different uppercase letters in the columns indicate a statistically significant difference by Tukey’s test (P< 0.05); Identical letters do not differ significantly from each other.

### Fatty Acid Composition in Seed Oils of Three Melon Cultivars by GC-FID

The results regarding the fatty acids present in the seed oils of three melon cultivars are presented in Table 2. For comparison, the compositional ranges established by ANVISA for olive, soybean, and sunflower oils are also included. Seventeen fatty acids were identified. The six most abundant fatty acids, considering the ranges observed among the cultivars, were, in descending order, linoleic acid (C18:2n6c) > oleic acid (C18:1n9c) > palmitic acid (C16:0) > stearic acid (C18:0) > cis-11,14,17-eicosatrienoic acid (C20:3n3) > α-linolenic acid (C18:3n3), with concentrations ranging from 47.50 to 65.18 g/100 g; 15.69 to 34.96 g/100 g; 7.80 to 10.48 g/100 g; 3.06 to 5.24 g/100 g; 0.13 to 0.58 g/100 g; and 0.10 to 0.18 g/100 g, respectively.

**Table 2:** Fatty Acid Profiles of Yellow, Piel de Sapo, and Cantaloupe Melon Seed Oils in Comparison with Reference Data from ANVISA,^19^ Firestone^20^ and Bailey’s Industrial Oil and Fat Products^21^ ^a^

	Melon seed oil (g/100 g)*			ANVISA (g/100 g)		
Fatty acids	Yellow melon	*Piel de Sapo* melon	Cantaloupe melon	Olive oil	Soybean oil	Sunflower oil
C14:0	tr	tr	tr	-	<0.5	<0.5
C15:0	tr	tr	tr	-	-	-
**C**16:0	8.09 ± 006^b^	7.80 ± 0.14^b^	10.48 ± 0.31^a^	7.5–20.0	9.5–13.3	3.0–10.0
C16:1	0.07 ± 0.01	0.07 ± 0.01	0.09 ± 0.02	0.3–3.5	tr	<1.0
C17:0	0.06 ± 0.01	tr	0.07 ± 0.00	<0.3	-	-
C17:1	tr	nd	nd	<0.3	-	-
**C**18:0	5.24 ± 0.01^a^	4.66 ± 0.05^b^	3.06 ± 2.65^ab^	0.5–5.0	3.0–6.1	1.0–10.0
**C**18:1**n9c**	25.45 ± 0.05^b^	34.96 ± 0.11^a^	15.69 ± 0.07^c^	63.3–81.5	19.0–30.0	14.0–35.0
**C**18:2**n6c**	55.98 ± 0.05^b^	47.50 ± 0.22^c^	65.18 ± 2.62^a^	5.1–15.5	44.0–62.0	55.0–75.0
**C**18:3**n3**	0.10 ± 0.08	0.11 ± 0.02	0.18 ± 0.04	0.3–0.9	4.0–11.0	<0.3
C20:1n9	0.10 ± 0.01	0.10 ± 0.00	tr	≤0.4	<1.0	<0.5
**C**20:3**n3**	0.15 ± 0.01	0.13 ± 0.01	0.58 ± 0.51	-	-	-
C20:5n3	0.05 ± 0.01	nd	tr	-	-	-
C21:0	0.07 ± 0.12	nd	nd	-	-	-
C22:0	tr	tr	nd	≤0.2	<0.5	<1.0
C22:6n3	tr	0.07 ± 0.06	0.12 ± 0.14	-	-	-
C24:0	tr	tr	tr	≤0.2	-	<0.5
SFA	13.61 ± 0.17	12.63 ± 0.10	13.67 ± 2.32	-	-	-
UFA	81.98 ± 0.17	82.96 ± 0.10	81.92 ± 2.32	-	-	-

a Values are expressed as mean ± standard deviation of analyses performed in triplicate. Different lowercase letters within the same column indicate significant differences among cultivars according to Tukey’s test (p < 0.05). SFA: saturated fatty acids; UFA: unsaturated fatty acids; nd: not detected; tr: Trace (concentration below 0.05% of total fatty acids). Myristic acid methyl ester (C14:0); pentadecanoic acid methyl ester (C15:0); palmitic acid methyl ester (C16:0); palmitoleic acid methyl ester (C16:1); heptadecanoic acid methyl ester (C17:0); cis-10-heptadecenoic acid methyl ester (C17:1); stearic acid methyl ester (C18:0); oleic acid methyl ester (C18:1n9c); linoleic acid methyl ester (C18:2n6c); α-linolenic acid methyl ester (C18:3n3); cis-11-eicosenoic acid methyl ester (C20:1n9); cis-11,14,17-methyl ester eicosatrienoic acid (C20:3n3); cis-5,8,11,14,17-eicosapentaenoic acid methyl ester (C20:5n3); heneicosanoic acid methyl ester (C21:0); behenic acid methyl ester (C22:0); cis-4,7,10,13,16,19-docosahexaenaic acid methyl ester (C22:6n3); lignoceric acid methyl ester (C24:0).

Analysis of the data in Table 2 shows that Cantaloupe melon presented the numerically highest total saturated fatty acid content (13.67 ± 2.32), however, the statistical data do not show a significant difference between the SFA values among the three types of melon. Regarding unsaturated fatty acids, Table 2 shows that the Piel de Sapo melon stands out for its high concentration of oleic acid (C18:1n9c), superior to that of the other melons, conferring benefits to cardiovascular health. The Cantaloupe melon, on the other hand, has a lower level of the monounsaturated fatty acid oleic acid (C18:1n9c) (15.69 ± 0.07). Regarding polyunsaturated fatty acids, Cantaloupe is the richest in linoleic acid (C18:2n6c) (65.18 ± 2.62), making it excellent for metabolic health. The Piel de Sapo melon showed higher levels of alpha-linolenic acid (C18:3n3), which may be a nutritional advantage for omega-3 supplementation. Despite differences in fatty acid composition, there was no significant difference in the total unsaturated fatty acids among the three types of melon studied. According to the parameters established by ANVISA^19^ for vegetable oils, the fatty acid profile of Cantaloupe melon and Yellow melon is similar to that of sunflower oil. The Piel de Sapo melon only falls short of the C18:2n6c level (47.50 ± 0.22 g/100 g), where the legislation establishes levels between 55.0–75.0 g/100 g.

The values of omega fatty acids (3, 6, and 9) found in melon seeds were compared with other conventional oils (sunflower, soybean, and olive), cited by Silva *et al.* ^18^ as shown in Figure 2. Low levels of omega-3, alpha-linolenic (C18:3n3) were found in melon seed oils, while soybean oil is richer in this compound (Figure 2). Higher levels of omega-6 - linoleic acid (C18:2n6c)—were found in Cantaloupe melon (65.18 ± 2.62 g/100 g). Yellow melon also showed high levels similar to those found in sunflower and soybean oil. Piel de Sapo melon has the highest value (34.96%) of omega-9, oleic (C18:1n9c), closest to sunflower oil.

**Figure 2:**
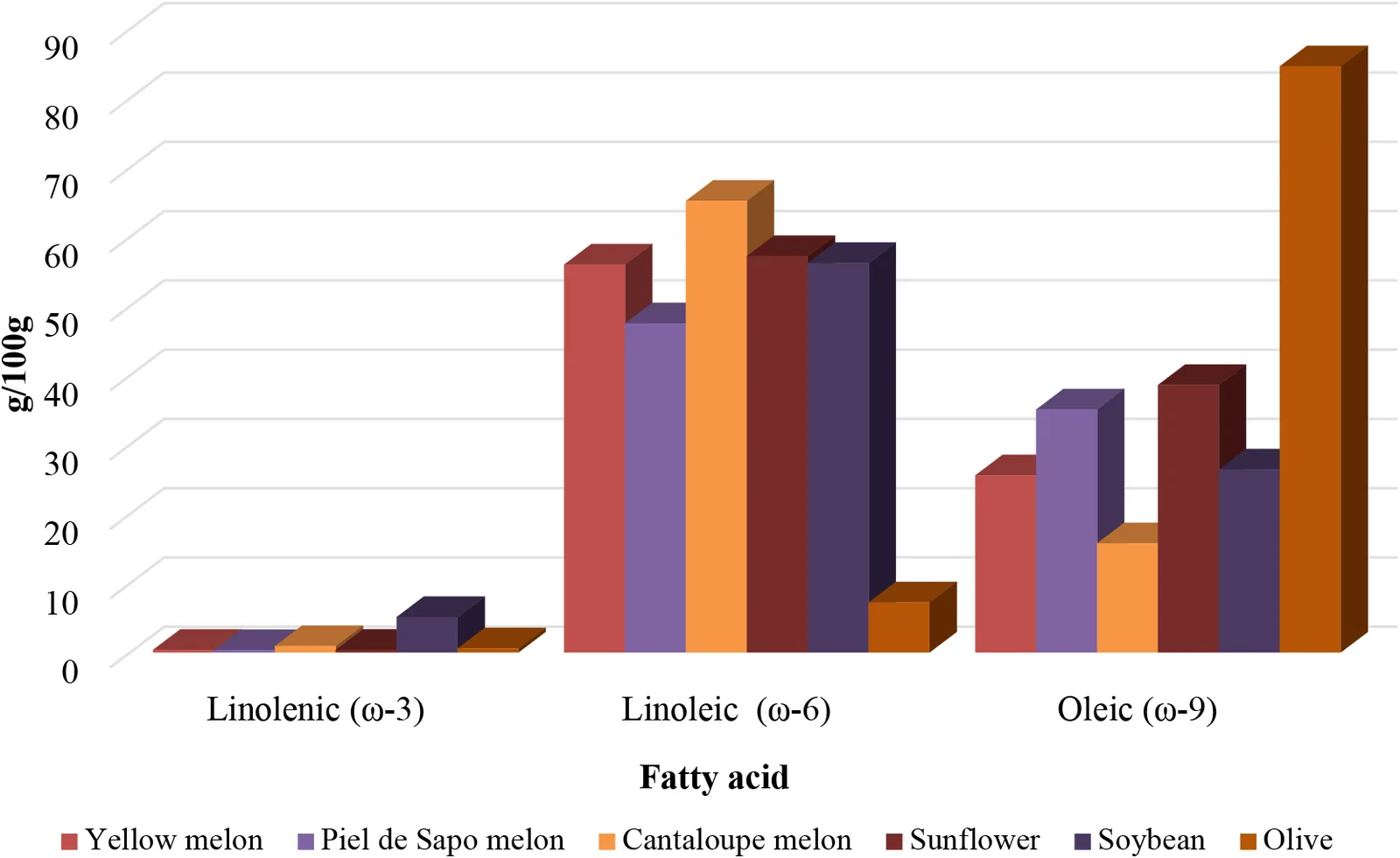
Comparison of fatty acids—omega 3 (*α- Linolenic* C18:3n3), omega 6 (linoleic C18:2n6c) and ômega 9 (Oleic C18:1n9c)—between different types of melon cultivars Yellow, *Piel de Sapo* and Cantaloupe and conventional oils. Silva *et al.* ^18^ sunflower, soybean and olive.

In addition to the absolute fatty acid concentrations, the balance between omega-6 and omega-3 fatty acids should be considered when evaluating the nutritional profile of these oils. Based on the concentrations of linoleic acid (LA; C18:2n6c) and α-linolenic acid (ALA; C18:3n3), the calculated LA/ALA ratios were approximately 560:1 for Yellow melon, 432:1 for *Piel de Sapo*, and 362:1 for Cantaloupe. These high ratios reflect the marked predominance of omega-6 fatty acids and the very low contribution of ALA in all three cultivars. Therefore, although α-linolenic acid was detected, the investigated melon seed oils should not be considered relevant dietary sources of omega-3 fatty acids; rather, they are predominantly omega-6-rich oils. A similar compositional pattern has been reported for other melon cultivars. Rabadán et al.^22^ reported linoleic acid contents ranging from approximately 51 to 69%, whereas α-linolenic acid remained between 0.14 and 0.26%. Likewise, Mallek-Ayadi et al.^23^ found 68.98% linoleic acid and only 0.20% α-linolenic acid in Maazoun melon seed oil.

The high proportion of polyunsaturated fatty acids observed in melon seed oils also highlights the importance of considering oxidative stability when evaluating their potential food applications. Because linoleic acid is particularly susceptible to lipid oxidation, fatty acid composition alone is insufficient to predict oil stability during processing and storage. Previous studies provide useful evidence in this regard. Mallek-Ayadi et al.^23^ reported a low peroxide value of 0.50 mequiv O_1_/kg and an oxidative induction time of approximately 7.20 h at 100 °C for Maazoun melon seed oil. Similarly, Rabadán et al.^22^ reported very low peroxide values for mechanically extracted Piel de Sapo seed oil, with no detectable peroxides immediately after extraction under either hydraulic or screw pressing conditions. However, these results also indicate that oxidative behavior may depend on cultivar, extraction conditions, processing temperature, and minor antioxidant compounds, and therefore cannot be directly extrapolated to the oils investigated in the present study.

The oxidative behavior of melon seed oil may also be influenced by naturally occurring bioactive compounds. Mallek-Ayadi et al.^23^ reported a total phenolic content of 22.63 mg gallic acid equivalents/100 g oil and identified several phenolic compounds, including amentoflavone, luteolin-7-O-glycoside, gallic acid, naringenin, and p-coumaric acid. In addition, found considerable concentrations of vitamin E in oils from different melon cultivars, ranging from approximately 232 to 531 mg/kg, with γ-tocopherol as the predominant isoform.^22^ These compounds are technologically relevant because endogenous antioxidants may contribute to protecting polyunsaturated lipids against oxidative deterioration. The broader literature also identifies melon seed oil as a source of tocopherols, phytosterols, and phenolic compounds, although substantial compositional variability among cultivars has been reported. Therefore, measurements of total phenolic content and antioxidant capacity using assays such as DPPH, FRAP, or related methods would be necessary to establish the antioxidant potential of the specific cultivars evaluated here rather than inferring it from literature values.

Physicochemical and sensory properties are also important when considering the suitability of unconventional oils for food formulations.^22,23^ The study also conducted a sensory evaluation with 60 untrained panelists and found that melon seed oil was generally accepted, although its overall appreciation remained lower than that of virgin olive oil.^23^ Furthermore, Silva et al.^24^ summarized previous evidence showing that melon seed oil has already been used as cooking oil in some regions and that blends of melon seed oil with peanut oil have been investigated in terms of physicochemical characteristics, oxidative stability, and organoleptic properties. Consequently, the term potential food application should be interpreted as a prospective use supported primarily by fatty acid composition and previous evidence for melon seed oils, rather than as a technologically validated application. Further testing is therefore required before proposing specific food formulations or culinary uses.

Thus, some general characteristics were observed in melon seed oils: they are rich in linoleic acid (omega 6), with the highest concentration found in Cantaloupe melon (65.18%). The Piel de Sapo melon is the richest in oleic acid-omega-9 (34.96%) among the melons investigated. Monounsaturated fatty acids, such as oleic acid, are known to reduce LDL cholesterol and increase HDL cholesterol. Low levels of omega-3 are found in the melon seed oil samples investigated, unlike soybean oils which show higher levels.

Principal Component Analysis (PCA) revealed the chemometric distinction between the seeds of the three melon varieties, with the first two components explaining 87.4% of the total variance of the data (Figure 3 A). The first principal component (PC1, 66.2%) represents the direct influence of the degree of unsaturation. The Cantaloupe variety was positioned on the positive axis, positively correlated with polyunsaturated fatty acids (C18:2n6c and C18:3n3) and palmitic acid (C16:0), while the Piel de Sapo variety clustered on the negative axis, characterized by a predominantly oleic profile (C18:1n9c). A strong vectorial antagonism was observed between C18:1n9c and C18:2n6c, indicating that variation in the relative proportions of oleic and linoleic acids was an important factor contributing to differentiation among the cultivars^25^ The second component (PC2, 21.2%) was decisive in distinguishing the Yellow melon variety, which, despite sharing the tendency toward higher levels of monounsaturated fats with Piel de Sapo, differed due to the greater contribution of stearic acid (C18:0).

**Figure 3:**
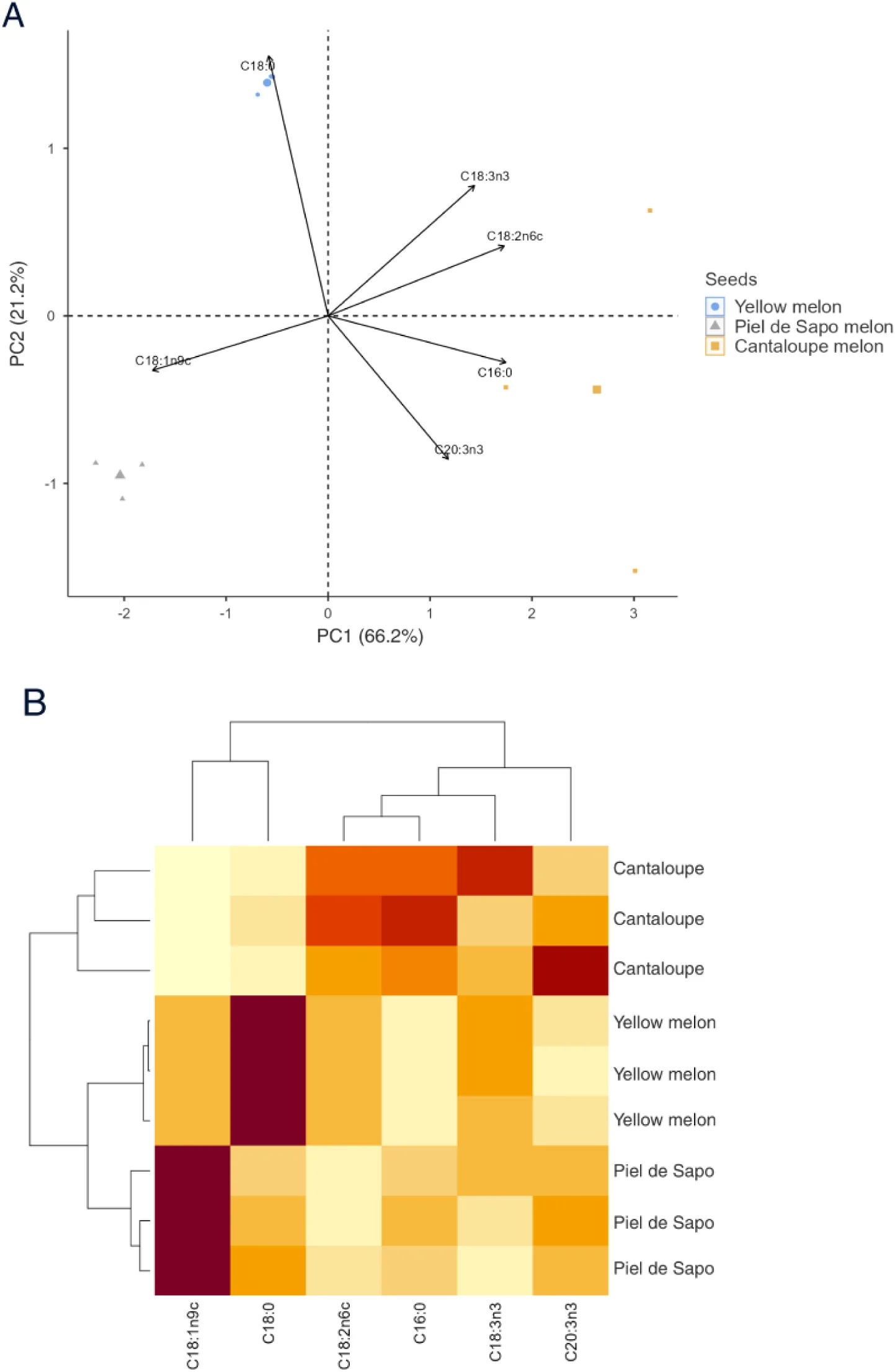
(A) Principal component analysis (PCA) and (B) heat map of the fatty acid composition of seed oils from three melon cultivars.

The lipid profile of the Piel de Sapo variety, strongly associated with the oleic acid vector (C18:1n9c) in the opposite quadrant, indicates distinct technological properties. The predominance of monounsaturated fatty acids (MUFAs) gives this oil superior oxidative stability compared to other varieties, since the lower presence of double bonds reduces susceptibility to lipid peroxidation and rancidity.^26^ This characteristic positions Piel de Sapo oil as a promising candidate for applications requiring greater thermal resistance, such as in industrial frying processes, where the stability of the ingredient is crucial for the shelf-life of the final product.

The heat map (Figure 3B) shows that the three melon varieties studied have similar fatty acid profiles, but with variations in the proportion of each compound. Saturated, monounsaturated, and polyunsaturated fatty acids are distributed in different intensities among the melons studied. The yellow melon shows a marked predominance of C18:0 (stearic acid), which is evidenced by the dark red color in the graph. The Piel de Sapo melon presents a more balanced proportion of saturated fatty acids (SFA) and saturated fatty acids (SFA) (represented by the dark yellow and light yellow colors) with higher concentrations of oleic acid (C18:1n9c), indicating a possible benefit for cardiovascular health. On the other hand, the Cantaloupe melon concentrates higher levels of C18:2n6c (linoleic acid) and C16:0 (palmitic acid). Alpha-linolenic acid (C18:3n3), an omega-3 fatty acid, is present in higher proportions in cantaloupe melon, making it nutritionally interesting for supplementation.

The valorization of melon processing by-products is strongly aligned with the concepts of biorefinery and the circular economy, retaining resources within the production chain to generate higher added value.^24^ The application of cold extraction using mechanical presses demonstrates significant industrial viability, as it allows for the production of high-quality virgin oils at affordable prices, avoiding the use of chemical solvents that would compromise the final quality of the product. Furthermore, the substantial variability in the fatty acid and bioactive compound profiles among different cultivars serves as a strategic factor for the targeted selection of raw materials.^22^ While certain cultivars stand out as potential sources of highly polyunsaturated oils rich in vitamin E (tocopherols and tocotrienols), their functional quality and suitability for developing new products are further reinforced by their phytochemical profile.^22–24^ Melon seed oil contains major phenolic compounds, such as amentoflavone and luteolin-7-O-glycoside,^23–26^ which play a crucial role in the oil’s oxidative stability, protecting polyunsaturated fatty acids against oxidation.

Therefore, understanding the specific variations of each cultivar allows for the optimization of processing strategies and drives the scalable application of these oils in the functional food industry. Regarding sustainability and market potential, oils extracted from melon seeds offer high potential for utilizing agricultural byproducts, since melons are widely consumed worldwide, generating tons of seeds as waste. One recommendation from the study would be to use the seeds of non-conforming melons for export (those that are not harvested in the fields and become fertilizer for the soil) for oil extraction, thus creating another source of income and adding value to the product.

## Conclusion

This study demonstrated a practical application of the circular economy and upcycling through the valorization of melon seeds, which are commonly considered agro-industrial by-products. The research used a low-cost, solvent-free method for extracting melon seed oil, thereby reducing the use of organic solvents and supporting more sustainable processing strategies.

The results obtained for the fatty acid profiles of oils from three melon cultivars showed the presence of 17 fatty acids, with six major compounds identified in descending order of abundance: linoleic acid (C18:2n6c) > oleic acid (C18:1n9c) > palmitic acid (C16:0) > stearic acid (C18:0) > cis-11,14,17-eicosatrienoic acid (C20:3n3) > α-linolenic acid (C18:3n3). Among the cultivars, Cantaloupe showed the highest linoleic acid content, whereas Piel de Sapo presented the highest oleic acid content.

The oils from the studied melon seeds contain fatty acids of nutritional and technological interest, particularly omega-6 and omega-9, suggesting their potential for food applications. Overall, the results highlight melon seeds as a promising unconventional raw material for the production of value-added oils, contributing to the reduction of agro-industrial waste and the valorization of the melon production chain. This study can contribute to contemporary gastronomy by transforming what was once waste into a high value-added product, expanding opportunities for new textures, flavors, and potential nutritional benefits.
